# Assessment of Lifestyle, Eating Habits and the Effect of Nutritional Education among Undergraduate Students in Southern Italy

**DOI:** 10.3390/nu15132894

**Published:** 2023-06-26

**Authors:** Fiorenzo Moscatelli, Antonella De Maria, Luigi Antonio Marinaccio, Vincenzo Monda, Antonietta Messina, Domenico Monacis, Giusi Toto, Pierpaolo Limone, Marcellino Monda, Giovanni Messina, Antonietta Monda, Rita Polito

**Affiliations:** 1 Department of Clinical and Experimental Medicine, University of Foggia, 71013 Foggia, Italy; fiorenzo400@gmail.com (F.M.); luigimarinaccio@me.com (L.A.M.); giovanni.messina@unifg.it (G.M.); 2 Department of Experimental Medicine, Section of Human Physiology, Unit of Dietetics and Sports Medicine, Università degli Studi della Campania “Luigi Vanvitelli”, 80131 Naples, Italy; antodemar82@gmail.com (A.D.M.); antonietta.messina@unicampania.it (A.M.); marcellino.monda@unicampania.it (M.M.); 3 Department of Movement Sciences and Wellbeing, University of Naples “Parthenope”, 80133 Naples, Italy; vincenzo.monda@uniparthenope.it; 4 Department of Humanities, Letters, Cultural Heritage, Educational Sciences, University of Foggia, 71100 Foggia, Italy; domenico.monacis@unifg.it (D.M.); giusi.toto@unifg.it (G.T.); 5 Department of Humanities, Telematic University “Pegaso”, 80143 Naples, Italy; pierpaolo.limone@unipegaso.it

**Keywords:** lifestyle, health, university students, nutritional education, correct nutrition, physical activity

## Abstract

Background: The years spent at university represent a critical period that can influence both the quality of lifestyle and the eating habits of subsequent adulthood, and also, in the long term, the health of the individual. The aim of this study was to investigate the lifestyle of university students living away from home. Methods: Each subject recruited for the study was given a questionnaire to obtain general information, eating habits and physical activity levels before (T0) and after six month of training seminars (T1). Blood pressure, body composition and questionnaire responses were investigated. Results: The main findings of this study are a significant decrement in blood pressure; an increment in physical activity practice; an increased number of subjects who pay attention to the calorific value of food and also an improvement in BIA parameters. Conclusions: In conclusion, this study demonstrated the challenges that university students face in leading a healthy lifestyle and caring for their nutritional needs, particularly when they are away from their families. No intervention specifically targets young adults, even though much emphasis is placed on the promotion of a healthy lifestyle based on a varied and balanced diet and sufficient exercise. Our study showed that it is possible to improve lifestyle through educational events aimed at making students aware of the health risks deriving from unhealthy lifestyles.

## 1. Introduction

The “healthy lifestyle” (HL) is a way of living, characterized by a methodical approach to behavior management across various domains [1]. Although there have been significant advancements in the prevention and management of chronic diseases, it is widely acknowledged that lifestyle factors, such as smoking, eating habits, stress management, and inactivity, play a significant role in the onset and progression of many chronic diseases [2]. Although clinicians may advise behavioral modifications such weight loss, quitting smoking, and exercising or engaging in physical activity (PA), convincing patients to follow this advice can be difficult [3]. However, even when efficient pharmaceutical, behavioral, and psychological therapies are available, it can be challenging to inspire people to start and continue lifestyle modifications. Adherence, which is also known as compliance [4], is described as “the degree to which a person’s behavior (in terms of taking drugs, following a diet, exercising, or adopting other lifestyle changes) agrees with medical or health advice” [5].The effectiveness of medical interventions can be negatively affected, and even completely eliminated, by non-adherence or by conflict between health practices and health advice. According to some estimates in general populations, up to 40% of patients do not follow medical advice to the letter, and when considerable lifestyle changes or difficult behavioral changes are necessary, this prevalence rate can reach 70% or higher [6]. Non-compliance can make treatment ineffective and erode trust in the healthcare practitioner. According to estimates, the total yearly costs of non-adherence to healthy lifestyles in the United States are in the hundreds of billions of dollars [7], with insufficient PA accounting for 10% of total health care spending [8].

A segment of the population particularly exposed to changes in lifestyle is university students who live outside their own families [9]. University students may be a group at biological and psychosocial risk, frequently engaging in unhealthy eating, insufficient resting habits, and substance addiction [10]. A shift in eating habits, such as a decrease in the intake of fresh or minimally processed meals and an increase in the consumption of ultra-processed goods, was brought on by changes in lifestyle during the previous several decades. A bad diet and other health issues, such as being overweight, are signs of new eating habits. The high frequency of stress in this demographic, emotional dysregulation with a lack of self-control, and identity concerns can all be linked to all these maladaptive behaviors among students. Modifying these unhealthy behaviors has the potential to be the focus of intervention while also enhancing academic success. Regarding this aspect, a recent multicenter study with 6222 college students reveals an intriguing trend of health-related behaviors across nations [11]. As observed in Romania, Lebanon, Turkey, and Croatia, students in some nations exhibited a cluster of unhealthier behaviors, such as a higher prevalence of smoking, skipping breakfast, and sleeping for shorter amounts of time on working days. Students from Italy and Spain, on the other hand, showed greater adherence to the Mediterranean diet, consumed more breakfast each day, smoked less, and slept for longer periods of time on working days. Furthermore, even in these young, generally healthy university students, we discovered a relationship between lifestyle and health outcomes, such as body mass index (BMI) and self-perceived health.

It is interesting to note that BMI was also positively correlated with total self-reported METs per week, which represents total physical activity. Since BMI is only a crude weight indicator for height and does not account for body composition, it is possible that the positive correlation between self-reported physical activity and BMI is caused by greater muscle mass [12]. Furthermore, the median METs per week for all countries suggested that the group overall had a moderate to high level of physical activity [13]. The practice of sports, even by overweight individuals, has been documented to contribute to less sedentary behavior in the past, but it has not been linked to healthy eating habits [14]. However, given that research typically shows a negative correlation between BMI and levels of physical activity [15], a more logical explanation would be that students with higher BMIs, even those within the normal range, were more involved in practicing their sports [15]. Moreover, only a marginal correlation between physical activity and BMI in non-obese patients has been shown previously [16], lending support to this latter hypothesis. Mobile phone use and perceived stress level, both of which were strongly correlated, were also positively associated with BMI. The reports from a recent systematic review and meta-analysis, showing that college students with mobile phone addiction were more likely to experience increased levels of anxiety, depression, and impulsivity as well as being more likely to have poor sleep quality [17], lend support to this association. The increasing popularity of smartphones has led to many problems due to excessive use. Excessive smartphone use can interfere with concentration at school or work and can cause physical difficulties, such as neck stiffness, blurred vision, wrist or back pain, and sleep disturbances. It can also reduce in-person social interaction and academic achievement and lead to relationship problems. Studies on smartphone use have shown that among smartphone users, 45.8% feel anxiety when they are not holding their smartphone, 27.1% spend too much time using their smartphone, and 22.6% have repeatedly attempted to reduce their smartphone use but have failed. Moreover, 21.0% of smartphone users reported difficulties with school or work due to excessive smartphone use. Further, these percentages were higher for individuals in their teens and twenties. Considering that addiction is a phenomenon characterized by tolerance, withdrawal symptoms, dependence, and social problems, the research described above suggests the concept of “smartphone addiction” [10]. In fact, data have revealed that the relationship between mobile phone addiction and numerous psychological and behavioral difficulties, including stress, anxiety and depression, might be mediated by interpersonal problems [18]. According to the interpersonal theory, people who are addicted to their mobile phones typically neglect real-world social networking, which leads to a lack of social resources and lower levels of physical activity [19]. In order to understand smartphone addiction, knowledge of risk and protective factors for such addiction is essential [20,21]. A person’s risk of obesity and cardiovascular problems may increase if they engage in little physical activity [22]. On the other hand, it has been hypothesized that physical activity can be used to reduce and regulate body fat [23]. More generally, regular exercise was shown to be an efficient way to lower a variety of health risk factors, particularly those connected to metabolic syndrome and cardiovascular diseases [24]. In order to maintain a suitable level of cardio-respiratory fitness, the American Academy of Sports Medicine specifically advises people to engage in at least 150 min per week of moderate intensity cardiovascular exercise and at least 75 min per week of strenuous intensity training. Also advised is resistance exercise 2–3 days per week [25]. Human health has long been a major concern, and research in the fields of medicine, biology, psychology, education, and social and philosophical inquiry have all focused on this issue. The ability to maintain health, prevent sickness, and lead a healthy lifestyle has always been correlated with the level of social development. It takes some instructional effort during sports and health and fitness activities to help children develop the understanding and belief that health is the highest value in a person and must be consistently kept. Given that one of the objectives set by the World Health Organization by 2020 is to promote a healthy lifestyle [26] and one of the key strategies for promoting health is to adopt a healthy lifestyle, it is important to design interventions to change unhealthy lifestyles and promote the dimensions and behaviors associated with health-promoting lifestyles. One of the approaches available for educational interventions in health promotion is the intervention mapping approach. This approach evaluates and intervenes on health-related problems from a problem solving and ecological perspective. Students are a large part of their community and the social capital of that community. On the one hand, studying the promotion of students’ lifestyles is effective in designing promotional interventions to promote healthy behaviors of these individuals. On the other hand, students with healthy lifestyles can become role models for other people in society [26].

Therefore, considering the situation of students who live outside their own households, and the importance that lifestyle has on health, the aim of this study was to evaluate the lifestyle and eating habits and the effect of nutritional education among undergraduate students in southern Italy.

## 2. Materials and Methods

### 2.1. Participant

For this study 80 subjects (age range: 18 to 28) were enrolled (in Table 1 are reported the anthropometric parameters and blood pressure values). However, the second evaluation was carried out on only 70 subjects, as 10 dropped out. All subjects were students at the University of Foggia and were recruited at ADISU Puglia (Agency for the Right to University Study) in cooperation with the Department of Clinical Experimental. All recruited subjects joined the “Lifestyle and Planet life: improving your life while saving the planet” project. To achieve this goal, a pilot study was conducted, which aimed to detect the physical activity level and lifestyle at the ADISU Residences. No exclusion criteria were applied for this investigation. Participants were provided with comprehensive information regarding the project and were assured that they were free to withdraw from the study at any time.

All students provided written informed consent before the beginning of the investigation and after having had a detailed explanation of the tests. The study was approved by the Institutional Ethics Committee of the University of Foggia on 22 May 2018, no.440/DS, and conducted according to the ethical principles of the Declaration of Helsinki.

### 2.2. Study Design

Each subject recruited for the study was given a questionnaire (Appendix A) to obtain general information, eating habits and physical activity levels before (T0) and after (T1) six months of training seminars. The training seminars were carried out via an electronic platform. The seminars focused on the following topics: nutrition and reduction of risk factors, physical activity and correct lifestyles. However, after the six-month training period, the number of subjects who underwent T1 measurements dropped to 70. Using Google forms (with the possibility of multiple answers), a questionnaire was administered under the supervision of researchers involved in the investigation. The questionnaire consisted of two multiple choice questions on physical activity levels, in which students could choose whether they practiced sports and, in the event of a positive answer, they could choose weekly attendance. Moreover, blood pressure was measured in each student at T0 and T1 by a team of doctors using a manual sphygmomanometer (Sfigmomanometer Aneroide Erka Perfect). Furthermore, before (T0) and after (T1) training seminars, each student was subjected to body composition analysis using a Quantum V Segmental Bioelectrical Impedence Analyzer (A-Wave, Santeramo in Colle, Bari, Italy). The topics covered during the training seminars concerned nutritional education, the composition of foods in terms of macronutrients and micronutrients, and the beneficial effects of physical activity, especially in the prevention and treatment of non-communicable diseases, such as obesity and cardiovascular diseases. Statistical analysis was performed using GraphPad 6 Software, Inc., Boston, MA, USA, for Windows, version 6.01, Dotmatics, R&D scientific. The data are presented as mean (M) ± standard deviation (SD), and statistical significance was set at *p* < 0.05. The Shapiro–Wilk test was used to check the normal distribution of variables. The paired samples *t*-test was performed to investigate the differences between T0 and T1 if data were normally distributed and the Wilcoxon test was performed if data were not normally distributed. The chi-square test was performed to investigate differences in questionnaire answers.

## 3. Results

**Figure 1:**
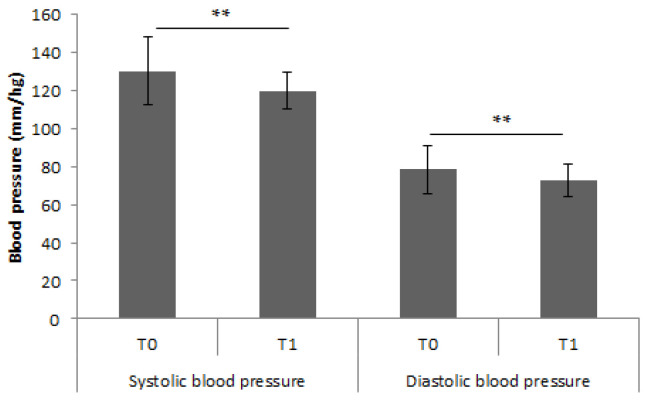
Differences in blood pressure between To and T1. ** indicates *p*-value < 0.01.

The first parameter analyzed was blood pressure. The results showed significant difference between T0 and T1. Systolic blood pressure decreased from T0 to T1 (130.40 ± 17.81 vs. 120.0 ± 9.58; *p* < 0.01), while diastolic blood pressure decreased from T0 to T1 (78.69 ± 12.60 vs. 72.89 ± 8.50, *p* < 0.01) (Figure 1).

Body composition, measured via bioelectrical impedance, showed significant differences between T0 and T1 (Table 2).

Regarding physical activity practice (Question 7), the results showed significant differences between T0 and T1; in fact, the number of the students that declared they practiced physical activity changed from yes 35, no 45 (T0) to yes 50, no 20 (T1) (Chi-square, df = 5.208, 1; z = 2.282; *p* < 0.05).

The percentage of subjects who tried to lose weight increased (Question 9). In fact, it went from 51% at T0 to 60% at T1; however, no significant differences emerged (Chi-square, df = 1.640, 1; z = 1.281; *p* > 0.05). Regarding question number 10, the percentage of subjects who declared that they paid attention to the caloric value of food increased from 63.75% to 78.0% (Chi-square, df = 4.104, 1; z = 2.026; *p* < 0.05).

**Figure 2:**
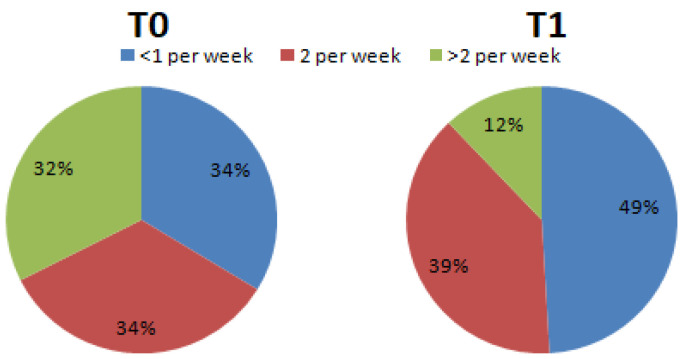
Percentage differences of subjects who declared they consumed sweets during the week (Chi-square, df = 12.69, 2; *p* < 0.01).

The portions of sweets consumed per week (Question 11) changed from T0 (<1 per week = 33.75%; 2 per week = 33.75%; >2 per week = 32.5%) to T1 (<1 per week = 49.1%; 2 per week = 38.9%; >2 per week 12%) (Figure 2).

**Figure 3:**
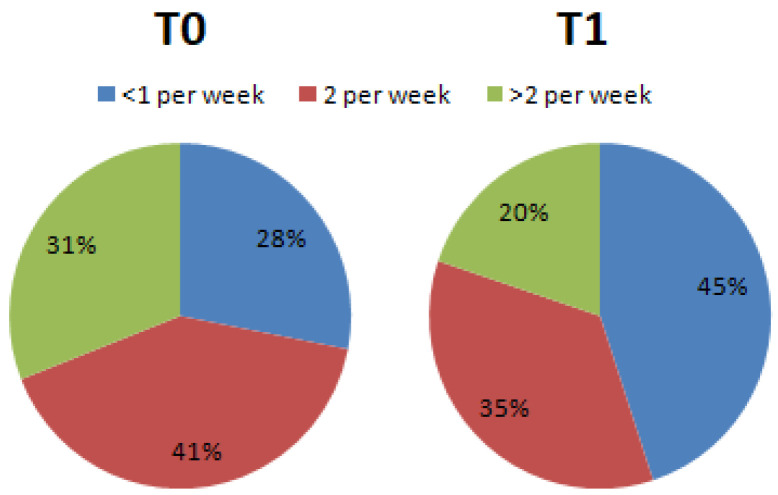
Percentage differences of subjects who declared they consumed red meat during the week (Chi-square, df = 6.805, 2; *p* < 0.05).

Regarding question number 13, servings of red meat consumed per week, the percentages changed from T0 (<1 per week = 28.1%; 2 per week = 40.9%; >2 per week = 31.0%) to T1 (<1 per week = 45.0%; 2 per week = 35.2%; >2 per week 19.8%) (Figure 3).

**Figure 4:**
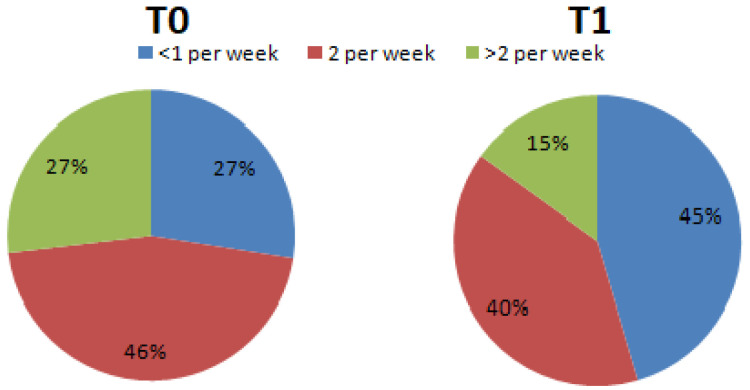
Percentage differences of subjects who declared they consumed processed meat during the week (Chi-square, df = 7.875, 2; *p* < 0.05).

Regarding question number 13, the percentage of processed meat consumed per week changed from T0 (<1 per week = 27.5%; 2 per week = 45.6%; >2 per week = 26.9%) to T1 (<1 per week = 45.5%; 2 per week = 39.5%; >2 per week = 15.0%) (Figure 4).

**Figure 5:**
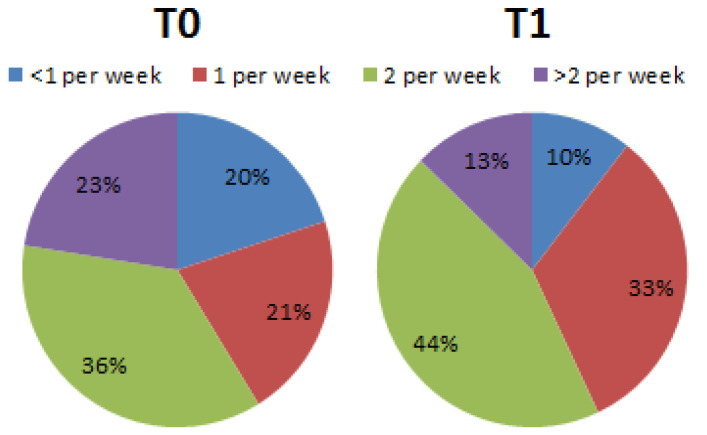
Percentage differences of subjects who declared they consumed legumes during the week (Chi-square, df = 9.353, 3; *p* < 0.05).

Regarding question number 14, the number of eggs consumed per week, the results showed no significant differences between T0 (<1 per week = 67.5%; 2 per week = 25.0%; >2 per week = 7.5%) and T1 (<1 per week = 67.5%; 2 per week = 25.5%; >2 per week = 7.0%). Regarding question number 15, the percentages of legumes consumed per week, the results showed significant differences between T0 (<1 per week = 20.0%; 1 per week = 21.25%; 2 per week = 36.25%; >2 per week = 22.5%) and T1 (<1 per week = 10.5%; 1 per week = 32.5%; 2 per week = 44.25%; >2 per week = 12.75%) (Figure 5).

**Figure 6:**
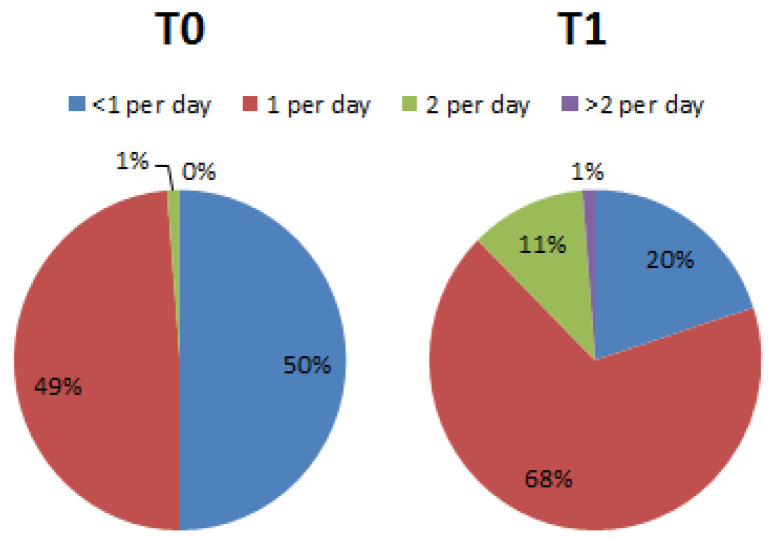
Percentage differences of subjects who declared they consumed fruits during the week (Chi-square, df = 25.33, 3; *p* < 0.001).

Regarding questions 16 (portions of white meat), 17 (fish or seafood) and 18 (dairy products consumed per day), no significant differences emerged between T0 and T1, while regarding question number 19, the percentage of fruit consumed per day, significant differences emerged between T0 (<1 per day = 50.0%; 1–2 per day = 48.75%; 3–4 per day = 1.25%; >4 per day = 0%) and T1 (<1 per day = 20.0%; 1–2 per day = 67.50%; 3–4 per day = 11.25%; >4 per day = 1,25%) (Figure 6).

Finally, regarding questions 20 (vegetables consumed per day), 21 (olive oil consumed per day), 22 (cereals consumed per day), 23 (glasses of water drunk every day), 24 (glasses of wine drunk every day), 25 (salt consumed every day), 26 (snacks consumed during meals), 27 (sugar intake) and 28 (hours spent watching TV a day), the results showed no significant differences between T0 and T1.

## 4. Discussion

The main purpose of this study was to evaluate the effect and impact of a training period focused on improving lifestyles. The main findings of this study are: (1) a significant decrement in blood pressure; (2) an increment in physical activity practice; (3) an increased number of subjects who paid attention to the calorific value of food; (4) a percentage decrease in subjects who declared they consumed sweets during the week; (5) a percentage decrease in subjects who declared they consumed red meat during the week; (6) a percentage decrease in processed meat consumed per week; (7) a percentage increase in subjects who declared they consumed legumes during the week; (8) a percentage increase in fruit consumed per day; (9) an improvement in BIA parameters. Our study, in addition to providing an overview of the lifestyle of students who live away from home, seems to show the effectiveness of training events as a tool to achieve improvement in lifestyles.

The results of our study are in line with previously published results by WHO [27]. In fact, given that the 2010 Global Recommendations on Lifestyle for Health state that one in four (27.5%) adults and more than three quarters (81%) of adolescents do not meet the recommendations for physical exercise and diet [28], there is an urgent need to increase the priority and investment in services that promote a healthy lifestyle [3]. These statistics also show that gender differences are significant and that participation levels have not increased globally over the past 20 years. Inequalities in involvement by age, gender, socioeconomic level and geography are also frequently seen in national data, which highlights the urgency of increasing financial support for health [4]. For current and future generations to remain healthy throughout their lifespans, proper diet is crucial. A balanced diet lowers the risk of chronic diseases while promoting healthy growth and development. Adults who follow a healthy diet have a lower risk of obesity, heart disease, type 2 diabetes, and several malignancies. They also live longer. People with chronic diseases can control their conditions and prevent complications by eating healthfully.

When there are no healthy options, consumers could select for items that are higher in calories and less nutritious. People from low-income areas and some racial and ethnic groups frequently do not have easy access to establishments that provide inexpensive healthier foods. Consequently, it is important to encourage people of all ages to eat healthily [29]. A healthy diet, according to the WHO (2016), should, for example, include a high intake of fruits, vegetables, and whole grains, as well as a low intake of saturated fats, salt, and processed carbohydrates [30]. Given that many healthy behaviors are formed and established during the transition from adolescence to young adulthood, this time may be a crucial moment for health promotion measures, including the promotion of good eating. Furthermore, excessive weight gain has been observed among young adults, particularly university students [31,32,33]. The transition from high school to university also coincides with new living conditions, which could cause eating habits to change [34]. However, few researchers have examined potential shifts in eating habits post matriculation.

Sport was the recreational activity students engaged in the most, despite doing so less frequently than the weekly average recommended for the maintenance of good health [14], which is consistent with other studies describing a lack of regular sport activities [14] and a decline in all forms of physical activity in correlation with the start of university [15]. According to an important investigation conducted in Southern Italy [11], students who live alone spend less time engaging in sports and other leisure activities overall [35].

From a nutritional standpoint, most respondents, particularly students living away from home, acknowledged that their eating habits had altered while they were attending college. It has been emphasized in other contexts how challenging it is for students to maintain appropriate eating routines [36]. The various factors that affect people’s eating choices include changes in lifestyle, the comfort and convenience of fast food, taste, their immediate physical and social environment, gender, attention to weight, and beliefs [37]. Other studies indicate that college students have adopted unhealthy eating habits, particularly with regard to low consumption of fruits and vegetables [38,39], milk and dairy products [40], fish [41], eggs, pulses [42], meat [43], sausages, and sweets [37,38,39,40].

College students from various backgrounds and with various dietary habits are affected by the difficulties of adopting a diet that complies with the Guidelines [32,37,40,41,42].

Although many students are influenced by the shift in eating patterns, those who lived away from their families are primarily impacted [43,44,45]. University students who lived with their parents consumed significantly more fruits, vegetables, legumes, and fish than those who did not live with their parents, which may be explained by the fact that they were not directly involved in meal planning and preparation while the family offered ongoing encouragement for making healthy food choices [46]. According to other studies [36,47], students who leave their families have a diet that deviates significantly from the ideal model of the Mediterranean diet in terms of their consumption of fruits, vegetables, pulses, and fish, while also consuming more ready-made foods and fries [48,49]. This is particularly true of students who are living on their own. These young people’s eating habits may be related to a variety of factors, including their first-ever complete independence [48], lack of expertise in meal planning and preparation, lack of time [49], or financial constraints that compel them to spend less on food [46].

The results of our study seem to suggest that attending university, especially away from the family, could play a role in the onset of unhealthy lifestyles, and that these could, at least in part, be modified through training events aimed at sensitizing this population during this moment of their life.

## 5. Conclusions

In conclusion, this study demonstrated the challenges that university students face in leading a healthy lifestyle and caring for their nutritional needs, particularly when they are away from their families. No intervention specifically targets young adults, even though much emphasis is placed on the promotion of a healthy lifestyle based on a varied and balanced diet and sufficient exercise.

Our study showed that it is possible to improve lifestyle through educational events aimed at making students aware of the health risks deriving from unhealthy lifestyles. However, although our study provided important results, there are some criticisms that will need to be investigated in the future. In particular, the sample should be increased to seek gender homogeneity; moreover, students who live in families alongside those who live in university residences should be encouraged to accurately evaluate the effect of the latter. In conclusion, it can be said that it would be appropriate to provide training and support programs in universities for students to improve their lifestyle. In fact, good nutrition is essential to keeping current and future generations healthy across the lifespan. Adults who eat a healthy diet live longer and have a lower risk of obesity, heart disease, type 2 diabetes, and certain cancers. New discoveries in health research happen every day, but one clear message remains consistent: optimal nutrition is imperative to human health. Research shows that an unhealthy diet is one of the major risk factors for a range of chronic diseases, including diabetes, cancer, cardiovascular diseases, and other conditions linked to obesity.

This is a study that comes from a pilot project that allowed us to obtain a statistical report on the university population of Foggia. Being a pilot project, this could be extended to other Apulian and national universities. Furthermore, because of the educational initiatives implemented during the project, improvements were highlighted both in the anthropometric parameters evaluated in the population and in the levels of physical activity, as well as through the effectiveness of the programmed training events, demonstrating that education on a healthy lifestyle and monitoring of body composition have a fundamental role in the conduct of a healthy lifestyle for the prevention of non-communicable diseases.

## Figures and Tables

**Table 1:** Anthropometric parameters and blood pressure values of subjects.

Parameters	Female	Male
Number (*n*)	49	21
Systolic pressure	124.25 mmHg ± 20.9	129.03 mmHg ± 19.8
Diastolic pressure	74.53 mmHg ± 13.1	76.92 mmHg ± 10.6
Height (cm)	162.2 ± 6.3	176.3 ± 8.2
Weight (kg)	61.9 ± 15.2	73.6 ± 13.5

**Table 2:** BIA analysis.

BIA Parameters	T0		T1		*p*-Value	
Weight (Kg)	66.18	±14.34	58.49	±9.54	0.0012	**
FFM%	72.31	±9.06	76.68	±6.59	0.1354	ns
FM%	27.69	±14.03	23.32	±6.34	0.0251	*
TBW%	54.26	±6.46	55.81	±4.63	0.0926	ns
ECW%	44.81	±3.43	45.68	±3.34	0.0654	ns
ICW%	54.53	±6.93	51.44	±12.08	0.0381	*
MM%	37.72	±6.89	38.45	±5.11	0.3144	ns
BCM%	27.04	±6.97	24.36	±6.32	0.0127	*
BM (kcal)	1533.94	±202.48	1456.64	±183.48	0.0130	*
BMI (kg/m²)	23.81	±4.39	21.85	±2.20	0.0022	**

* Indicates *p*-value < 0.05; ** Indicates *p*-value < 0.01.

## Data Availability

The data presented in this study are available on request from the corresponding author.

## Appendix A

	***n* (%)**	***n* (%)**	***n* (%)**	***n* (%)**
Question 1: Sex	Male	Female		
Question 2: Age				
Question 3: Smoke	Yes	No		
Question 4: COVID Vaccination	Yes	No		
Question 5: COVID Positivity	Yes	No		
Question 6: Gastrointestinal disorders/Other disease	Yes	No		
Question 7: Do you practice physical activity?	Yes	No		
Question 8: If yes, how many times a week?	<1 time per week	1–2 times per week	2–3 times per week	>3 times per week
Question 9: Have you been trying to lose weight in the past three months? If not: Have you tried to avoid gaining weight?	No attempt to either lose or avoid gaining weight in the past three months	Attempts to both lose weight and avoid gaining weight in the past three months for reasons related to body shape or weight		
Question 10: Do you pay close attention to the calorific value of food?	Yes	No		
Question 11: How many portions of sweets do you consume per week? (Chocolates (1 portion = 3 g) biscuits and similar)	<1 serving per week	2 servings per week	>2 servings per week	
Question 12: How many servings of red meat do you eat per week? (Beef, pork, lamb, 1 portion 100–150 g)	<1 serving per week	2 servings per week	>2 servings per week	
Question 13: How many servings of processed meat do you consume per week? (sausages and similar; 1 portion = 50 g)	<1 serving per week	2 servings per week	>2 servings per week	
Question 14: How many eggs do you eat per week? (1 serving = 1 egg)	<1 serving per week	2 servings per week	>2 servings per week	
Question 15: How many servings of legumes do you consume per week? (Lentils, beans, peas, chickpeas (1 serving = 1 plate or 150 g)	<1 serving per week	1 serving per week	2 servings per week	>2 servings per week
Question 16: How many portions of white meat do you eat per week? (Poultry, rabbit—1 serving = 150 g)	<1 serving per week	2 servings per week	>2 servings per week	
Question 17: How many servings of fish or seafood do you eat per week? (White/fatty fish (1 serving = 100–150 g), canned fish (1 serving = 1 can or 50 g), seafood (1 serving = 200 g))	<1 serving per week	2 servings per week	>2 servings per week	
Question 18: How many dairy products do you consume per day? (Milk—1 portion = 200 mL milk, two yogurts, 1 portion of spreadable cheese)	<1 serving per day	2 servings per day	>2 servings per day	
Question 19: How much fruit do you consume per day? (All fruit and fresh fruit juices—1 serving = 150–200 g)	<1 serving per day	1–2 servings per day	3–4 servings per day	>4 servings per day
Question 20: How many servings of vegetables do you consume per day? (1 portion = 150–200 g)	<1 serving per day	1–2 servings per day	>2 servings per day	
Question 21: How many tablespoons of olive oil do you consume per day (cooking or salad dressing)? (Olive oil, extra virgin olive oil—1 serving = 1 tablespoon)	<1 serving per day	1–2 servings per day	>2 servings per day	
Question 22: How many servings of cereals do you consume per day? (White and wholemeal bread—1 serving = 40 g, half sandwich, cereals—1 serving = 1 plate of rice, pasta or 40 g of breakfast cereal)	<1 serving per day	1–2 servings per day	>2 servings per day	
Question 23: Do you drink more than 6 glasses of water or at least one cup of tea a day? (Water or tea—1 glass serving)	Yes	No		
Question 24: Do you drink wine every day with meals? (White/red wine (1 serving = 1 glass of wine))	<1 serving per day	1–2 servings per day	>2 servings per day	
Question 25: Do you limit adding salt to meals?	Yes	No		
Question 26: Do you usually use nibbling between meals? Do you consume snacks between meals?	Yes	No		
Question 27: Do you limit your sugar intake in drinks? (including sugary drinks)	Yes	No		
Question 28: How many hours do you spend watching TV a day?	<1 h per day	1 h a day	2 h a day	>2 h a day

## References

[1] Stonerock G.L., Blumenthal J.A. (2017). Role of Counseling to Promote Adherence in Healthy Lifestyle Medicine: Strategies to Improve Exercise Adherence and Enhance Physical Activity. Prog. Cardiovasc. Dis..

[2] Pratt M., Perez L.G., Goenka S., Brownson R.C., Bauman A., Sarmiento O.L., Hallal P.C. (2015). Can Population Levels of Physical Activity Be Increased? Global Evidence and Experience. Prog. Cardiovasc. Dis..

[3] Sallis R., Franklin B., Joy L., Ross R., Sabgir D., Stone J. (2015). Strategies for Promoting Physical Activity in Clinical Practice. Prog. Cardiovasc. Dis..

[4] Mifsud J.L., Galea J., Garside J., Stephenson J., Astin F. (2020). Motivational interviewing to support modifiable risk factor change in individuals at increased risk of cardiovascular disease: A systematic review and meta-analysis. PloS ONE.

[5] McDonald H.P., Garg A.X., Haynes R.B. (2002). Interventions to Enhance Patient Adherence to Medication Prescriptions: Scientific Review. JAMA.

[6] Martin L.R., Williams S.L., Haskard K.B., Dimatteo M.R. (2005). The Challenge of Patient Adherence. Ther. Clin. Risk Manag..

[7] Krienke R. (2005). Adherence to Medication. N. Engl. J. Med..

[8] Carlson S.A., Fulton J.E., Pratt M., Yang Z., Adams E.K. (2015). Inadequate Physical Activity and Health Care Expenditures in the United States. Prog. Cardiovasc. Dis..

[9] Cena H., Porri D., De Giuseppe R., Kalmpourtzidou A., Salvatore F.P., El Ghoch M., Itani L., Kreidieh D., Brytek-Matera A., Pocol C.B. (2021). How Healthy Are Health-Related Behaviors in University Students: The HOLISTic Study. Nutrients.

[10] Lyzwinski L.N., Caffery L., Bambling M., Edirippulige S. (2019). The Mindfulness App Trial for Weight, Weight-Related Behaviors, and Stress in University Students: Randomized Controlled Trial. JMIR mHealth uHealth.

[11] Cena H., Tesone A., Niniano R., Cerveri I., Roggi C., Turconi G. (2013). Prevalence Rate of Metabolic Syndrome in a Group of Light and Heavy Smokers. Diabetol. Metab. Syndr..

[12] Mertens E., Deriemaeker P., Van Beneden K. (2021). Adjustments in Food Choices and Physical Activity during Lockdown by Flemish Adults. Nutrients.

[13] Haddad C., Hallit R., Akel M., Honein K., Akiki M., Kheir N., Obeid S., Hallit S. (2020). Validation of the Arabic Version of the ORTO-15 Questionnaire in a Sample of the Lebanese Population. Eat. Weight Disord.-Stud. Anorex. Bulim. Obes..

[14] Hemmingsson E., Ekelund U. (2007). Is the Association between Physical Activity and Body Mass Index Obesity Dependent?. Int. J. Obes..

[15] Li Y., Li G., Liu L., Wu H. (2020). Correlations between Mobile Phone Addiction and Anxiety, Depression, Impulsivity, and Poor Sleep Quality among College Students: A Systematic Review and Meta-Analysis. J. Behav. Addict..

[16] Taheri S., Lin L., Austin D., Young T., Mignot E. (2004). Short Sleep Duration Is Associated with Reduced Leptin, Elevated Ghrelin, and Increased Body Mass Index. PLoS Med..

[17] Remskar M., Western M.J., Maynard O.M., Ainsworth B. (2022). Exercising body but not mind: A qualitative exploration of attitudes to combining physical activity and mindfulness practice for mental health promotion. Front. Psychol..

[18] Zaccagni L., Barbieri D., Gualdi-Russo E. (2014). Body Composition and Physical Activity in Italian University Students. J. Transl. Med..

[19] Sacheck J.M., Kuder J.F., Economos C.D. (2010). Physical Fitness, Adiposity, and Metabolic Risk Factors in Young College Students. Med. Sci. Sports Exerc..

[20] Wagner A., Dallongeville J., Haas B., Ruidavets J.B., Amouyel P., Ferrières J., Simon C., Arveiler D. (2012). Sedentary Behaviour, Physical Activity and Dietary Patterns Are Independently Associated with the Metabolic Syndrome. Diabetes Metab..

[21] Garber C.E., Blissmer B., Deschenes M.R., Franklin B.A., Lamonte M.J., Lee I.M., Nieman D.C., Swain D.P. (2011). Quantity and Quality of Exercise for Developing and Maintaining Cardiorespiratory, Musculoskeletal, and Neuromotor Fitness in Apparently Healthy Adults: Guidance for Prescribing Exercise. Med. Sci. Sports Exerc..

[22] WHO (2010). Global Recommendations on Physical Activity for Health.

[23] Guthold R., Stevens G.A., Riley L.M., Bull F.C. (2018). Worldwide Trends in Insufficient Physical Activity from 2001 to 2016: A Pooled Analysis of 358 Population-Based Surveys with 1·9 Million Participants. Lancet. Glob. Health.

[24] Hilger J., Loerbroks A., Diehl K. (2017). Eating Behaviour of University Students in Germany: Dietary Intake, Barriers to Healthy Eating and Changes in Eating Behaviour since the Time of Matriculation. Appetite.

[25] Beard J.R., Officer A., de Carvalho I.A., Sadana R., Pot A.M., Michel J.-P., Lloyd-Sherlock P., Epping-Jordan J.E., Peeters G.M.E.E.G., Mahanani W.R. (2016). The World Report on Ageing and Health: A Policy Framework for Healthy Ageing. Lancet.

[26] Mensink G.B.M., Schienkiewitz A., Haftenberger M., Lampert T., Ziese T., Scheidt-Nave C. (2013). Overweight and Obesity in Germany: Results of the German Health Interview and Examination Survey for Adults (DEGS1). Bundesgesundheitsblatt. Gesundheitsforschung. Gesundh..

[27] Nelson Laska M., Larson N.I., Neumark-Sztainer D., Story M. (2010). Dietary Patterns and Home Food Availability during Emerging Adulthood: Do They Differ by Living Situation?. Public Health Nutr..

[28] Lupi S., Bagordo F., Stefanati A., Grassi T., Piccinni L., Bergamini M., De Donno A. (2015). Assessment of Lifestyle and Eating Habits among Undergraduate Students in Northern Italy. Ann. Ist. Super. Sanita.

[29] Cluskey M., Grobe D. (2009). College Weight Gain and Behavior Transitions: Male and Female Differences. J. Am. Diet. Assoc..

[30] Driskell J.A., Kim Y.-N., Goebel K.J. (2005). Few Differences Found in the Typical Eating and Physical Activity Habits of Lower-Level and Upper-Level University Students. J. Am. Diet. Assoc..

[31] Nicklas T.A., Baranowski T., Cullen K.W., Berenson G. (2001). Eating Patterns, Dietary Quality and Obesity. J. Am. Coll. Nutr..

[32] Debate R.D., Topping M., Sargent R.G. (2001). Racial and Gender Differences in Weight Status and Dietary Practices among College Students. Adolescence.

[33] Stefanikova Z., Sevcikova L., Jurkovicova J., Sobotova L., Aghova L. (2006). Positive and Negative Trends in University Students’ Food Intake. Bratisl. Lek. Listy.

[34] Colić Barić I., Satalić Z., Lukesić Z. (2003). Nutritive Value of Meals, Dietary Habits and Nutritive Status in Croatian University Students According to Gender. Int. J. Food Sci. Nutr..

[35] Hercberg S., Preziosi P., Galan P., Deheeger M., Papoz L., Dupin H. (1991). Dietary Intake of a Representative Sample of the Population of Val-de-Marne; III. Mineral and Vitamin Intake. Rev. Epidemiol. Sante Publique.

[36] Skemiene L., Ustinaviciene R., Piesine L., Radisauskas R. (2007). Peculiarities of Medical Students’ Nutrition. Medicina.

[37] Huang T.T.K., Harris K.J., Lee R.E., Nazir N., Born W., Kaur H. (2003). Assessing Overweight, Obesity, Diet, and Physical Activity in College Students. J. Am. Coll. Health.

[38] Anding J.D., Suminski R.R., Boss L. (2001). Dietary Intake, Body Mass Index, Exercise, and Alcohol: Are College Women Following the Dietary Guidelines for Americans?. J Am. Coll. Health.

[39] Mammas I., Bertsias G., Linardakis M., Moschandreas J., Kafatos A. (2004). Nutrient Intake and Food Consumption among Medical Students in Greece Assessed during a Clinical Nutrition Course. Int. J. Food Sci. Nutr..

[40] Racette S.B., Deusinger S.S., Strube M.J., Highstein G.R., Deusinger R.H. (2005). Weight Changes, Exercise, and Dietary Patterns during Freshman and Sophomore Years of College. J. Am. Coll. Health.

[41] Di Palma D., Tafuri D. (2016). Special needs and inclusion in sport management: A specific literature review|Posebne potrebe i uključenje u sportskom menadžmentu: Specifičan pregled literature. Sport Sci..

[42] Mazzeo F., Santamaria S., Monda V., Messina G., Monda M. (2016). Dietary supplements use in competitive and non-competitive boxer: An exploratory study. Biol. Med..

[43] Gaetano R., Domenico T., Gaetano A. (2015). Physical activity and its relation to body and ludic expression in childhood Mediterranean. J. Soc. Sci..

[44] Monda M., Viggiano A., Vicidomini C., Tafuri D., De Luca B. (2009). Expresso coffee increases parasympathetic activity in young, healthy people. Nutr. Neurosci..

[45] Sirangelo I., Borriello M., Liccardo M., Scafuro M., Russo P., Iannuzzi C. (2021). Hydroxytyrosol Selectively Affects Non-Enzymatic Glycation in Human Insulin and Protects by AGEs Cytotoxicity. Antioxidants.

[46] Kresić G., Kendel Jovanović G., Pavicić Zezel S., Cvijanović O., Ivezić G. (2009). The Effect of Nutrition Knowledge on Dietary Intake among Croatian University Students. Coll. Antropol..

[47] Arroyo Izaga M., Rocandio Pablo A.M., Ansotegui Alday L., Pascual Apalauza E., Salces Beti I., Rebato Ochoa E. (2006). Diet Quality, Overweight and Obesity in University Students. Nutr. Hosp..

[48] Zykova S.N., Storhaug H.M., Toft I., Chadban S.J., Jenssen T.G., White S.L. (2015). Cross-Sectional Analysis of Nutrition and Serum Uric Acid in Two Caucasian Cohorts: The AusDiab Study and the Tromsø Study. Nutr. J..

[49] Pan Y.L., Dixon Z., Himburg S., Huffman F. (1999). Asian Students Change Their Eating Patterns after Living in the United States. J. Am. Diet. Assoc..

